# Feasibility of an LGBTQ+ Public Health Surveillance Platform in Kentucky: A Brief Report on Mental Health Signals

**DOI:** 10.3390/healthcare13202626

**Published:** 2025-10-19

**Authors:** Keith J. Watts, Sydney P. Howard, Missy Spears, Carolyn Lauckner, Rachel H. Farr, Glenn Means, Justin X. Moore

**Affiliations:** 1 College of Social Work, University of Kentucky, Lexington, KY 40508, USA; keithjwatts@uky.edu (K.J.W.); glenn.means@uky.edu (G.M.); 2 Center for Health, Engagement, and Transformation, College of Medicine, University of Kentucky, Lexington, KY 40536, USA; sydney.howard@uky.edu; 3 Queer Kentucky, Louisville, KY 40206, USA; missy@queerkentucky.com; 4 C. S. Mott Department of Public Health, College of Human Medicine, Michigan State University, Flint, MI 48502, USA; clauck@msu.edu; 5 Department of Psychology, University of Kentucky, Lexington, KY 40508, USA; rachel.farr@uky.edu; 6 Markey Cancer Center, University of Kentucky, Lexington, KY 40536, USA; 7 Department of Internal Medicine, College of Medicine, University of Kentucky, Lexington, KY 40536, USA

**Keywords:** LGBTQ+, minority stress, social capital, mental health, survey implementation, Kentucky

## Abstract

**Background/Objectives**: Robust, state-level LGBTQ+ health surveillance is scarce in Kentucky, limiting evidence-based healthcare planning and policy. We aimed to evaluate the feasibility and early public-health utility of a community-partnered annual survey and compare selected mental health stressors between Kentucky and non-Kentucky respondents. **Methods**: We conducted a cross-sectional online survey (13 April–15 July 2024) developed with a statewide LGBTQ+ nonprofit. Recruitment occurred via organizational channels and community events. A content warning preceded the survey, which was administered via Qualtrics. Data quality was screened using reCAPTCHA. We assessed feasibility metrics including recruitment and completion rates. Mental health stressors were captured with a six-item scale. Group differences were estimated with Welch’s *t*-tests. **Results**: Of 3852 survey starts, 1559 were retained as analyzable completes (completion rate: 40.47%), with 78.7% residing in-state. Initial analysis revealed a significant divergence in mental health patterns: while Kentucky participants reported lower stress regarding their personal mental health, they reported significantly higher stress stemming from socio-political issues like homophobia and transphobia compared to out-of-state respondents. **Conclusions**: An annual, community-partnered surveillance platform is a feasible strategy for generating actionable mental health signals relevant to healthcare. These findings will inform targeted outreach and guide health system partnerships to enhance LGBTQ+-affirming care in Kentucky.

## 1. Introduction

According to the Movement Advancement Project (2024), 3.4% of Kentucky adults identify as LGBTQ+ (Lesbian, Gay, Bisexual, Transgender, Queer/Questioning, and other sexual identities) which comprises 4% of Kentucky’s workforce, and 26% of those individuals are raising children [1]. Almost half of LGBTQ+ youth (and almost 60% of transgender and nonbinary youth) in Kentucky report seriously considering suicide in the past year [2].

These concerning mental health statistics can be understood through the lens of the Minority Stress Model, which posits that elevated health disorders in LGBTQ+ populations result from chronic stress due to social stigma, discrimination, and a hostile socio-political environment [3]. For LGBTQ+ people of color, this stress is often intersectional, involving experiences of racism within LGBTQ+ spaces and heterosexism within their racial/ethnic communities, which can manifest as daily microaggressions [4]. Our survey was designed to capture key tenets of this model, including direct measures of everyday and major discrimination experiences and their perceived impact on mental health. This data gap is particularly acute given the rise of anti-LGBTQ+ state laws [5], the mental health impacts of which were a specific focus of our survey instrument. From a healthcare systems perspective, the absence of routine sexual orientation and gender identity data capture constrains clinical screening for these stressors and the allocation of mental health resources. Furthermore, even when services are advertised as being LGBTQ-specific, a significant majority offer no tangible, tailored programming, creating a critical gap in affirming care [6].

To address this gap, we partnered with a diverse, LGBTQ+-led nonprofit to create an annual survey that leverages its established trust and network. Therefore, the primary objectives of this brief report are twofold: (1) to describe the feasibility and methodology of our novel, community-partnered recruitment strategy, and (2) to present a preliminary analysis comparing mental health stressors between LGBTQ+ individuals in Kentucky and those outside the state, thereby providing crucial data for future healthcare interventions.

## 2. Materials and Methods

### 2.1. Participants and Recruitment

This study was a cross-sectional survey design using convenience and snowball sampling. To maximize participant reach the survey was distributed and promoted through the non-profit’s channels and at community events between 13 April and 15 July 2024. The online Qualtrics survey was preceded by a content warning advising participants of questions related to substance use, mental health, and sexual behaviors. Participation was incentivized through a random drawing in which 25 participants received $100 gift cards. The project received Institutional Review Board approval (protocol 94156), and electronic informed consent was obtained from all participants. Data was stored and protected by the university’s firewalls and VPNs.

### 2.2. Measures

The online survey included demographic information, health behaviors, and several scales related to the Minority Stress Model. The primary outcome, mental health stressors, was assessed using a custom-developed list of 22 items asking, “How frequently do you experience stress or anxiety related to the following issues?” on a 5-point Likert-type scale from “Never” (0) to “Always” (4). The six items used in this manuscript’s analysis were: “My mental health”, “Threats of violence against LGBTQ+ spaces”, “Anti-LGBTQ+ hate crimes”, “Homophobia”, “white supremacists”, and “Transphobia”, were selected for this preliminary analysis as they directly pertain to socio-political minority stressors central to the study’s hypothesis. The full instrument also included comprehensive measures of discrimination and an 18-item LGBTQ+ Belongingness Scale, which were not part of this preliminary analysis.

### 2.3. Statistical Analysis

**Figure 1:**
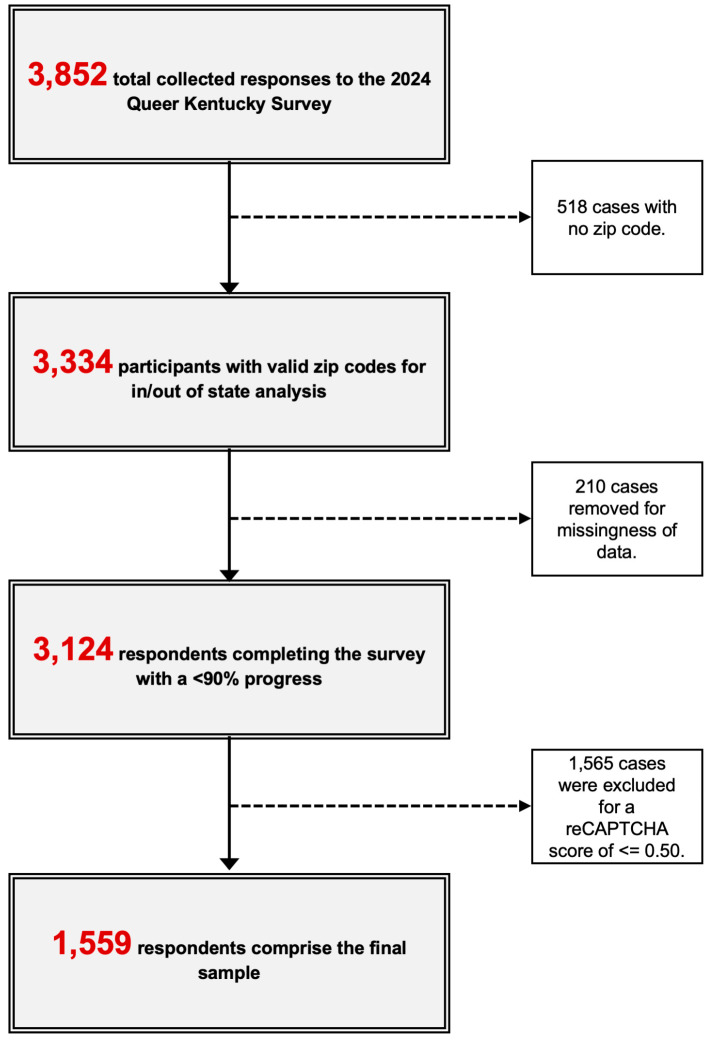
Flowchart of participant inclusion for analytic sample.

Statistical analyses were conducted using SPSS Statistics Version 29. For the purpose of describing the reach of the survey to its target audience, we created a dichotomous LGBTQ+ identification variable. Those selecting Gay, Lesbian, Bisexual, Pansexual, Queer, Asexual, Autosexual, or Demisexual were coded as LGBTQ+. Those selecting “Heterosexual/Straight” were coded as non-LGBTQ+, while “Questioning or unsure” and “Prefer not to answer” were excluded from this specific dichotomous analysis to avoid misclassification. Responses with a <0.5 ReCAPTCHA score and missing zip codes were excluded from the sample (Figure 1). We compared group characteristics using Chi-Square tests for categorical responses and Fisher’s Exact tests categorical responses with expected cell sizes less than five. We then examined group differences in mental health items. First, Levene’s Test of Equality of Variances was conducted to assess homogeneity of variance across groups. The Kolmogorov–Smirnov test was used to evaluate the normality of distribution for each response item. Results indicated that variances between group item means were not equal; therefore, the assumption of equal variances was violated. Consequently, nonparametric Mann–Whitney U tests were conducted, and the associated *p*-values are reported. To account for multiple comparisons, a Bonferroni correction was applied in Table 1 (adjusted *p*-value threshold = 0.05/12 = 0.004) when comparing participant characteristics. For Table 2, Bonferroni-adjusted confidence intervals were used to maintain the overall type I error rate (1 − α/6 = 99.17% CI).

## 3. Results

### 3.1. Feasibility and Reach

**Figure 2:**
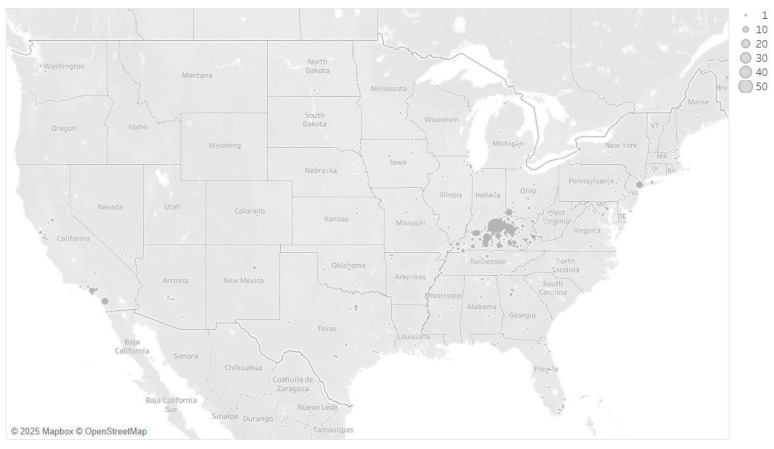
Geographic distribution of respondents created in Tableau Legend shows the density of respondents by zip code.

The platform demonstrated strong feasibility. Over a three-month field period, the survey yielded 1559 analyzable responses from 3852 starts, for a completion rate of 40.47%. This resulted in a recruitment rate of approximately 111 completes per week. The majority of participants (83.45%) resided in Kentucky (see Figure 2 for the geographic distribution of all respondents).

### 3.2. Sample Characteristics

The final sample was predominantly white (85.82%), identified as Gay (59.40%), and held a Bachelor’s degree (68.95%). Within Kentucky, 94.08% of respondents identified as LGBTQ+. Significant demographic and health behavior differences were observed between Kentucky and out-of-state participants, particularly in PrEP usage (73.87% vs. 45.74%) and HIV/AIDS testing (76.48% vs. 49.22%) (*p* < 0.001 for both). A full summary is available in Table 1.

### 3.3. Mental Health Stressors

Overall, the sample reported high levels of stress across several domains. A little over 68% of all respondents reported experiencing stress or anxiety about their mental health at least “sometimes,” with approximately 67% reporting stress about anti-LGBTQ+ hate crimes and 64% reporting stress about transphobia (Table 2). Kentuckians reported significantly higher stress related to external, socio-political threats, including white supremacists (*p* = 0.003) and transphobia (*p* = 0.001) (Table 2).

## 4. Discussion

The success of this project demonstrates that a community-partnered surveillance platform is a feasible and effective method for engaging LGBTQ+ populations in Kentucky. The central finding suggests a paradoxical nature of mental health stressors: lower stress about personal mental health, but higher stress from societal threats.

This paradox may reflect the dual nature of social capital, a concept related to the notion that relationships and community ties have important implications for well-being [7]. It is plausible that LGBTQ+ individuals in Kentucky have developed resilient, tight-knit local support networks—a form of bonding social capital—that buffer against internal distress. Research suggests this protective effect of community connectedness on mental health is often strongest for racial and ethnic minorities within the LGBTQ+ community, for whom these spaces may be particularly vital [8]. The concept of community as a source of support and validation aligns with the fundamental human need to belong, which is crucial for individual psychosocial wellbeing [9,10]. This hypothesis is supported by the survey instrument itself, which included a comprehensive 18-item LGBTQ Belongingness Scale designed to measure these very aspects of community connection. However, this same community embeddedness also makes them more acutely aware of the external minority stressors posed by discriminatory policies prevalent in the state [3]. This aligns with research showing that a strong sense of belonging, while often beneficial, can also heighten vigilance to discrimination, potentially increasing stress and its associated health consequences [11].

These findings must also be understood within Kentucky’s unique sociopolitical landscape. Research shows that living in states with more restrictive, less inclusive policies is associated with significantly worse mental health and higher rates of substance use among LGBTQ+ adults [12]. The patterns observed in our sample are therefore likely shaped by a structural environment that is further compounded by regional challenges, such as the disproportionate impact of the substance use crisis in Appalachian counties [13].

### Limitations

This study has several limitations. Our use of convenience and snowball sampling may limit generalizability, and the cross-sectional design prevents causal inference. Because the sample was community-connected and unweighted, estimates may be subject to coverage bias, particularly with respect to rurality, race/ethnicity, and sexual orientation/gender identity (SOGI). The sample was also predominantly white and college educated. Additionally, the selection of items for analysis was conducted post hoc and without initial multiplicity control, which may increase the risk of Type I error. Future analyses of this rich dataset should examine the relationship between the specific measures of everyday and major discrimination and the mental health outcomes reported here. Furthermore, it is important to acknowledge that the stressors measured are often compounded for LGBTQ+ people of color, who navigate racism within LGBTQ spaces and homophobia within their racial/ethnic communities, creating a dual burden of minority stress [4,14,15].

## 5. Conclusions

In a turbulent political climate, supporting the LGBTQ+ community through research is crucial. This study demonstrates a feasible model for collecting vital health data and reveals that for LGBTQ+ Kentuckians, stressors are often external and systemic. The findings underscore the need for public health interventions and policies that not only support individual mental health but also work to dismantle the structures of homophobia and transphobia that create a hostile environment.

## Figures and Tables

**Table 1:** Summary of population characteristics among 1559 survey respondents participating in the 2024 survey.

	In Kentucky (Column %) *n* = 1301	Out of State (Column %) *n* = 258	Overall *n* = 1559	*p*-Value
**Age (categorical)** **(*n* = 1515)**				
18–21	60 (4.71)	18 (7.47)	78 (5.15)	
22–25	146 (11.46)	69 (28.63)	215 (14.19)	
26–30	143 (11.22)	85 (35.27)	228 (15.05)	
31–35	444 (34.85)	41 (17.01)	485 (32.01)	
36–40	433 (33.99)	13 (5.39)	446 (29.44)	
41–50	30 (2.35)	12 (4.98)	42 (2.77)	
51–60	12 (0.94)	2 (0.83)	14 (0.92)	
61–64	2 (0.16)	1 (0.41)	3 (0.21)	
65+	4 (0.31)	0 (0.00)	4 (0.26)	<0.001
**Education**				
Some high school or less	7 (0.54)	5 (1.94)	12 (0.77)	
High school diploma or GED	53 (4.07)	21 (8.14)	74 (4.75)	
Some college, but no degree	119 (9.15)	44 (17.05)	163 (10.46)	
Associates or technical degree	66 (5.07)	27 (10.47)	93 (5.97)	
Bachelor’s degree	947 (72.79)	128 (49.61)	1075 (68.95)	
Graduate or professional degree	108 (8.30)	31 (12.02)	139 (8.92)	
Prefer not to say	1 (0.08)	2 (0.78)	3 (0.18)	<0.001
**Ethnicity**				
Spanish, Hispanic, or Latino	114 (8.76)	120 (46.51)	234 (15.00)	
Non-Hispanic/Latino	1187 (91.24)	138 (53.49)	1325 (85.00)	<0.001
**Gender Identity**				
Transgender Woman	41 (3.15)	15 (5.81)	56 (3.59)	
Transgender Man	52 (4.00)	28 (10.85)	80 (5.13)	
Gender Non-Conforming	10 (0.77)	2 (0.78)	12 (0.77)	
Genderqueer or fluid	57 (4.38)	14 (5.43)	71 (4.55)	
Two-spirit	2 (0.15)	5 (1.94)	7 (0.45)	
Nonbinary	46 (3.54)	16 (6.20)	62 (3.98)	
Other	5 (0.38)	4 (1.55)	9 (0.58)	
Cisgender Woman	155 (11.91)	77 (29.84)	232(14.88)	
Cisgender Man	926 (71.18)	84 (32.56)	1010 (64.79)	<0.001
**Sexual Orientation**				
Asexual	7 (0.54)	7 (2.71)	14 (0.90)	
Autosexual	0 (0.00)	3 (1.16)	3 (0.19)	
Bisexual	88 (6.76)	43 (16.67)	131(8.40)	
Demisexual	4 (0.31)	0 (0.00)	4 (0.26)	
Gay	869 (66.79)	57 (22.09)	926 (59.40)	
Heterosexual/Straight	66 (5.07)	68 (26.36)	134 (8.60)	
Lesbian	70 (5.38)	29 (11.24)	99 (6.35)	
Other	5 (0.38)	3 (1.16)	8 (0.51)	
Pansexual	43 (3.31)	6 (2.33)	49 (3.14)	
Prefer not to answer	2 (0.15)	6 (2.33)	8 (0.51)	
Queer	143 (10.99)	64 (13.18)	177 (11.35)	
Questioning or unsure	4 (0.31)	2 (0.78)	6 (0.38)	<0.001
**Race**				
American Indian/Native American or Alaska Native	7 (0.54)	11 (4.26)	18 (1.15)	
Asian	5 (0.38)	52 (20.16)	16 (1.03)	
Black or African American	33 (2.54)	52 (20.16)	85 (5.45)	
Native Hawaiian or Other Pacific Islander	31 (2.38)	5 (1.94)	36 (2.31)	
White or Caucasian	1178 (90.55)	160 (62.02)	1338 (85.82)	
More than one race	42 (3.23)	12 (4.65)	54 (3.46)	
Other	4 (0.31)	4 (1.55)	8 (0.51)	
Prefer not to say	1 (0.08)	3 (1.16)	4 (0.26)	<0.001
**Income**				
Less than $25,000	64 (4.92)	33 (12.79)	97 (6.22)	
$25,000–$49,999	126 (9.68)	54 (20.93)	180 (11.55)	
$50,000–$74,999	143 (10.99)	62 (24.03)	205 (13.15)	
$75,000–$99,999	469 (36.05)	51 (19.77)	520 (33.35)	
$100,000–$149,999	425 (32.67)	41 (15.89)	466 (29.89)	
$150,000+	64 (4.92)	12 (4.65)	76 (4.87)	
Prefer not to say	10 (0.77)	5 (1.94)	15 (0.96)	<0.001
**General Health**				
Excellent	305 (23.44)	34 (13.18)	339 (34.12)	
Good	467 (35.90)	117 (45.35)	584 (37.46)	
Average	446 (34.28)	86 (33.33)	532 (34.12)	
Poor	76 (5.84)	18 (6.98)	94 (6.03)	
Terrible	7 (0.54)	3 (1.16)	10 (0.64)	<0.001
**Current Smoker**				
Yes	611 (46.96)	136 (52.71)	747 (47.92)	
No	390 (53.04)	122 (47.29)	812 (52.08)	<0.001
**History of Cancer**				
Yes	52 (4.00)	35 (13.57)	87 (5.58)	
No	1249 (96.00)	223 (86.43)	1472 (94.42)	<0.001
**PrEP Usage**				
Yes	961 (73.87)	118 (45.74)	1079 (69.21)	
No	340 (26.13)	140 (54.26)	480 (30.79)	<0.001
**HIV/AIDS Test**				
Yes	995 (76.48)	127 (49.22)	1122 (71.97)	
No	306 (23.52)	131 (50.78)	437 (28.03)	<0.001

After Bonferroni-adjustment, *p*-value is significant < 0.004.

**Table 2:** Comparison of mean scores of mental health questions using Mann–Whitney U test. Comparing mental health between in-state and out-of-state participants among Queer Kentucky participants in 2024.

	Kentucky Respondents No. Reporting ≥ 2 (%)	Out-of-State Respondents No. Reporting ≥ 2 (%)	Kentucky Respondents *M* (*SD*)	Out-of-State Respondents *M* (*SD*)	Overall Item *M* (*SD*)	*Cohen’s d*	*99.17 CI*	*p*-Value
**My mental health** (*n* = 1557)	856 (65.90)	156 (60.47)	2.14 (1.31)	2.33 (1.03)	2.17 (1.26)	1.26	−0.28, −0.16	0.06
**Threats of violence against LGBTQ+ spaces** (*n* = 1556)	821 (63.15)	181 (70.70)	2.05 (1.32)	2.12 (1.14)	2.06 (1.29)	1.29	−0.19, 0.08	0.51
**Anti-LGBTQ+ hate crimes** (*n* = 1554)	863 (66.38)	177 (69.69)	2.11 (1.32)	2.12 (1.32)	2.11 (1.30)	1.30	−0.14, 0.13	0.98
**Homophobia** (*n* = 1553)	845 (65.15)	162 (63.28)	2.09 (1.35)	1.95 (1.24)	2.07 (1.33)	1.33	−0.03, 0.24	0.09
**White supremacists** (*n* = 1554)	857 (66.08)	162 (63.04)	2.15 (1.34)	1.91 (1.27)	2.11 (1.33)	1.33	0.05, 0.31	0.008 *
**Transphobia** (*n* = 1555)	849 (65.41)	145 (56.42)	2.13 (1.33)	1.75 (1.23)	2.07 (1.32)	1.31	0.16, 0.43	0.001 *

Participants were asked, “How frequently do you experience stress or anxiety related to the following issues?” among 1559 respondents. *n* varies due to missing responses on individual items. * Significant two-sided, Bonferroni adjustment for six total comparisons, *p* < 0.008.

## Data Availability

The data presented in this study are available on request from the corresponding author. The data are not publicly available due to privacy restrictions.

## Abbreviations

The following abbreviations are used in this manuscript:

PrEP	Pre-Exposure Prophylaxis
LGBTQ+	Lesbian, Gay, Bisexual, Transgender, Queer/Questioning, and other sexual identities
